# Association between high-density lipoprotein cholesterol and reversion to normoglycemia from prediabetes: an analysis based on data from a retrospective cohort study

**DOI:** 10.1038/s41598-023-50539-w

**Published:** 2024-01-02

**Authors:** Zihe Mo, Haofei Hu, Yong Han, Changchun Cao, Xiaodan Zheng

**Affiliations:** 1 Department of Physical Examination, DongGuan Tungwah Hospital, Dongguan, 523000 Guangdong Province China; 2 Department of Nephrology, Shenzhen Second People’s Hospital, The First Affiliated Hospital of Shenzhen University, Shenzhen, 518000 Guangdong Province China; 3 Department of Emergency, Shenzhen Second People’s Hospital, The First Affiliated Hospital of Shenzhen University, No.3002, Sungang West Road, Futian District, Shenzhen, 518000 Guangdong Province China; 4 Department of Rehabilitation, Shenzhen Second People’s Hospital, Shenzhen Dapeng New District Nan’ao People’s Hospital, No. 6, Renmin Road, Dapeng New District, Shenzhen, 518000 Guangdong Province China; 5 Department of Neurology, Shenzhen Samii Medical Center, The Fourth People’s Hospital of Shenzhen, No. 1 Jinniu West Road, Shijing Street, Pingshan District, Shenzhen, 518000 Guangdong Province China

**Keywords:** Pre-diabetes, Diseases, Endocrinology

## Abstract

The available evidence on the connection between high-density lipoprotein cholesterol (HDL-C) levels and the reversion from prediabetes (Pre-DM) to normoglycemia is currently limited. The present research sought to examine the connection between HDL-C levels and the regression from Pre-DM to normoglycemia in a population of Chinese adults. This historical cohort study collected 15,420 Pre-DM patients in China who underwent health screening between 2010 and 2016. The present research used the Cox proportional hazards regression model to investigate the connection between HDL-C levels and reversion from Pre-DM to normoglycemia. The Cox proportional hazards regression model with cubic spline functions and smooth curve fitting was employed to ascertain the nonlinear association between HDL-C and reversion from Pre-DM to normoglycemia. Furthermore, a set of sensitivity analyses and subgroup analyses were employed. Following the adjustment of covariates, the findings revealed a positive connection between HDL-C levels and the likelihood of reversion from Pre-DM to normoglycemia (HR 1.898, 95% CI 1.758–2.048, P < 0.001). Furthermore, there was a non-linear relationship between HDL-C and the reversion from Pre-DM to normoglycemia in both genders, and the inflection point of HDL-C was 1.540 mmol/L in males and 1.620 mmol/L in females. We found a strong positive correlation between HDL-C and the reversion from Pre-DM to normoglycemia on the left of the inflection point (Male: HR 2.783, 95% CI 2.373–3.263; Female: HR 2.217, 95% CI 1.802–2.727). Our sensitivity analysis confirmed the robustness of these findings. Subgroup analyses indicated that patients with SBP < 140 mmHg and ever smoker exhibited a more pronounced correlation between HDL-C levels and the reversion from Pre-DM to normoglycemia. In contrast, a less robust correlation was observed among patients with SBP ≥ 140 mmHg, current and never smokers. This study provides evidence of a positive and nonlinear association between HDL-C levels and the reversion from Pre-DM to normoglycemia in Chinese patients. Implementing intensified intervention measures to control the HDL-C levels of patients with Pre-DM around the inflection point may substantially enhance the likelihood of regression to normoglycemia.

## Introduction

Diabetes mellitus (DM) is a multifaceted and often asymptomatic chronic ailment that can lead to severe consequences, potentially resulting in premature mortality^1^. Prediabetes is characterized by a blood glucose level exceeding the "normal" range but falling below the diagnostic threshold for type 2 diabetes mellitus (T2DM)^2^. According to the International Diabetes Federation's 2017 estimation, 374 million adults worldwide were afflicted with prediabetes, and this number is projected to rise to 548 million by 2045, constituting 8.4% of the adult population^3^. Approximately 86 million adults in the United States, accounting for 37% of the population, are affected by Pre-DM^4^. Similarly, in China, the prevalence of Pre-DM stands at approximately 35.7% among adults^5^. Moreover, Pre-DM elevates the risk of developing T2DM and increases the likelihood of cardiovascular disease and microvascular complications^6–8^. However, it is important to acknowledge that certain individuals with Pre-DM do not progress to diabetes but remain in the prediabetic stage. Furthermore, a notable percentage of individuals, ranging from 20 to 50%, may revert to normoglycemia^9–11^. Previous studies have indicated that even a temporary return to normal blood glucose levels in individuals with Pre-DM is related to a significantly decreased chance of developing T2DM^12^. Therefore, it is crucial to highlight the significant clinical advantages of transitioning from Pre-DM to normoglycemia. The primary objective of screening and treating Pre-DM should be to restore normoglycemia.

The current emphasis in clinical research is primarily directed toward understanding the progression of diseases, thereby overshadowing the significance of identifying factors that contribute to the regression of Pre-DM toward normoglycemia. However, it is crucial to prioritize investigating these factors as they can offer valuable insights into preventive measures and actionable targets for sustaining public health initiatives. Regrettably, the number of studies conducted to ascertain the rate of reversion from Pre-DM to normoglycemia and the associated contributing factors remains limited. Previous epidemiological evidence suggests that the reversion from Pre-DM to normoglycemia is linked to various factors, including insulin secretion, age, dyslipidemia, obesity, and baseline fasting glucose^4,13–16^. Most scholars widely recognize high-density lipoprotein cholesterol (HDL-C) as a protective factor against diabetes. Research has demonstrated that an elevation in HDL-C levels is inversely related to the likelihood of transitioning from Pre-DM to diabetes^17–19^. However, there is a shortage of research on the correlation between HDL-C levels and the reversion from Pre-DM to normoglycemia. A study with a limited sample size from a German national cohort identified female and elevated HDL-C levels as determinants for the reversion from Pre-DM to normoglycemia^20^. Similarly, another cohort research with a slightly larger sample size demonstrated a positive connection between heightened HDL-C levels and the probability of the reversion from Pre-DM to normoglycemia^21^. Regrettably, prior investigations concerning the connection between HDL-C levels and the reversion from Pre-DM to normoglycemia lacked subgroup analyses or examination of the non-linear connection between these variables. Furthermore, previous studies investigating the connection between HDL-C levels and the reversion from Pre-DM to normoglycemia suffered from limited sample sizes. Additionally, the association between HDL-C levels and reversion to normoglycemia remains largely unexplored among Chinese adults. Therefore, this research aimed to examine the correlation between HDL-C levels and the reversion from Pre-DM to normoglycemia in a substantial sample of the Chinese population, utilizing a publicly accessible database.

## Research objects and methods

### Data source

The primary data were acquired at no cost from DATADRYAD (www.datadryad.org). The dataset was derived from a previously published study by Chen et al.^22^. This article is made available under the Creative Commons Attribution Non-Commercial license, allowing individuals to modify, remix, create, and share derivative works for non-commercial purposes, provided proper credit is given to the author and source.

### Study population

The Rich Healthcare Group Review Board authorized the initial research^22^. This secondary analysis does not require ethical approval. The present research followed the Declaration of Helsinki. All procedures, including the Declarations section declarations, followed relevant laws and conventions.

**Figure 1:**
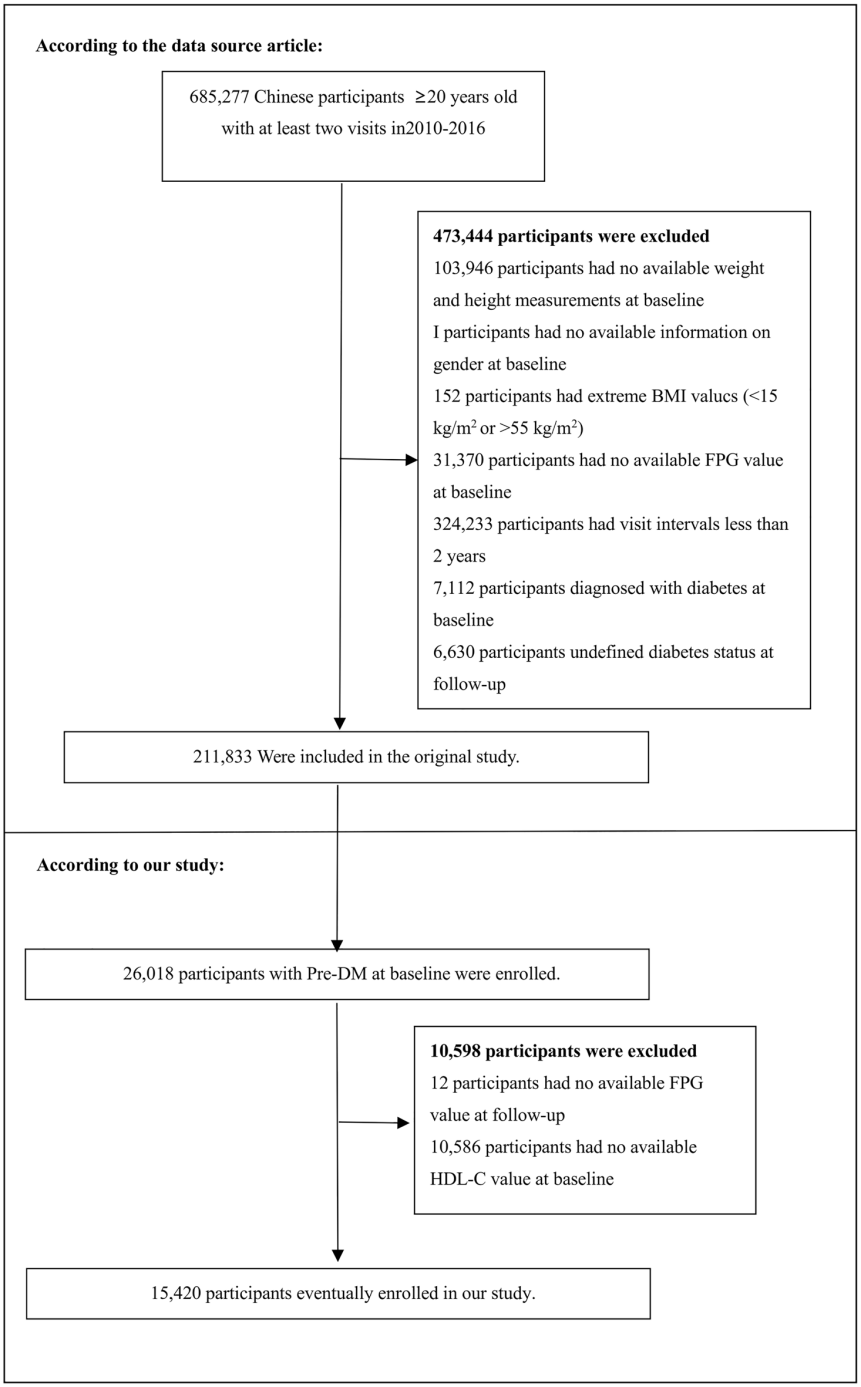
Study population.

The initial research included 685,277 individuals aged 20 and above from the Rich Healthcare Group in China who had undergone at least two health examinations. A total of 473,444 subjects were excluded from the research due to not meeting the specified exclusion criteria. The exclusion criteria for this research were as follows: (1) individuals with follow-up intervals < 2 years; (2) individuals with extreme body mass index (BMI) values (< 15 kg/m^2^ or > 55 kg/m^2^); (3) individuals lacking information on fasting plasma glucose (FPG), weight, sex, and height at baseline; (4) individuals with DM at the time of inclusion; and (5) individuals with unknown DM status during follow-up. Finally, the initial research involved 211,833 individuals^22^. A total of 26,018 subjects with Pre-DM were entered into the present research. We excluded individuals with missing FPG information at follow-up (n = 12) and HDL-C information at baseline (n = 10,586). Ultimately, the current research included a total of 15,420 participants. The selection process for participants is depicted in Fig. 1.

### Data collection

In the initial study, researchers of professional standing employed standardized questionnaires to collect baseline data pertaining to alcohol consumption, tobacco usage, and familial predisposition to diabetes. Trained personnel conducted measurements of subjects' blood pressure, height, and weight. BMI is obtained by dividing weight (kg) by the square of height (m). At each visit, venous blood samples were obtained following a minimum 10-h fasting period. The FPG and total cholesterol (TC), aspartate aminotransferase (AST), low-density lipoprotein cholesterol (LDL-C), blood urea nitrogen (BUN), alanine aminotransferase (ALT), high-density lipoprotein cholesterol (HDL-C), triglyceride (TG), and serum creatinine (Scr) were quantified using a Beckman 5800 autoanalyzer. The definition of prediabetes was impaired fasting glucose levels (FPG: 5.6–6.9 mmol/L). According to the American Diabetes Association (ADA) criteria, the definition of Pre-DM is characterized by impaired fasting glucose levels falling within the range of 5.6 to 6.9 mmol/L for FPG^23^. HDL-C measured at baseline is the independent variable. The dependent variable is the reversion to normoglycemia from Pre-DM discovered during the follow-up. Participants' return dates for one or more physical exams were used to determine when normoglycemia would return.

### Outcome measures

Reversion to normoglycemia was determined according to no self-reported diabetic events and FPG less than 5.6 mmol/L at follow-up^23,24^.

### Missing data processing

In our study, the number of subjects with missing data on TG, SBP, TC, DBP, AST, LDL-C, BUN, ALT, smoking status, drinking status, and Scr were 2 (0.013%), 5 (0.032%), 2 (0.013%), 5 (0.032%), 8295 (53.794%), 28 (0.182%), 364 (2.361%), 37 (0.240%), 10,675 (69.228%), 10,675 (69.228%) and 116 (0.752%), respectively. In order to reduce the volatility brought on by missing variables, which make it impossible to fully reflect the target sample's statistical effectiveness throughout the modeling phase, this study employed multiple imputations for missing data. The imputation model includes TG, SBP, TC, DBP, AST, LDL-C, BUN, ALT, smoking status, drinking status, sex, Scr, age, family history of diabetes, and BMI. It also employed linear regression and ten iterations. The missing data analysis strategies used the missing-at-random (MAR) assumptions^25,26^.

### Statistical analysis

We used R software 3.6.1 and Empower Stats (R) version 2.2 for data analysis.

The participants were separated into four groups (Q1 ≤ 1.120, 1.120 < Q2 ≤ 1.310, 1.310 < Q3 ≤ 1.510, Q4 > 1.510) based on their HDL-C quartiles. For continuous data with Gaussian distributions, the means and standard deviations were provided; for data with skewed distributions, the medians were supplied; and for categorical data, the frequencies and percentages were reported. To look for differences between various HDL-C groups, we employed the Kruskal–Wallis H test (data with skewed distribution), χ^2^ (categorical data), or the One-Way ANOVA test (data with normal distribution). We expressed the incidence rates in terms of cumulative incidence and person-years. Using the Kaplan–Meier approach, survival and cumulative event rate comparisons were made. Additionally, using the log-rank test, we analyzed the Kaplan–Meier hazard ratios (HR) of adverse events.

After conducting collinearity screening, we employed univariate and multivariate Cox proportional hazards regression models to investigate the association between HDL-C and the reversion from Pre-DM to normoglycemia. This analysis included three models: Model 1, which did not involve any adjusted covariates; Model 2, which adjusted for minimal covariates (adjusted family history of diabetes, DBP, age, sex, drinking status, SBP, BMI, and smoking status); and Model 3, which incorporated full covariate adjustments (adjusted DBP, smoking status, SBP, BMI, gender, age, drinking status, FPG, AST, BUN, ALT, LDL-C, Scr, TG, and family history of diabetes). The study recorded effect sizes (HR) and their corresponding 95% confidence intervals (CI). According to the findings of univariate analysis, literature reports, and clinical experience, adjustments were made to account for potential confounding factors. Furthermore, the final multivariate Cox proportional hazards regression equation excluded TC due to its collinearity with other variables (Table S1).

In order to ascertain the dependability of the results, a sequence of sensitivity analyses was conducted. Prior research has indicated a significant association between BMI and age with the reversion from Pre-DM to normoglycemia^21,27^. A sensitivity analysis was conducted on subjects with a BMI < 25 kg/m^2^. Furthermore, another sensitivity analysis was performed by excluding subjects with age ≥ 60 years. To validate the reliability of the findings, the continuity covariate was also integrated into the equation as a curve utilizing a generalized additive model (GAM). In addition, we conducted E-value calculations to assess the potential influence of unmeasured confounding on the connection between HDL-C levels and the reversion from Pre-DM to normoglycemia^28^.

Additionally, we employed a Cox proportional hazards regression model with cubic spline functions and smooth curve fitting to address the non-linear association between HDL-C levels and the reversion to normoglycemia from Pre-DM. Moreover, a two-piecewise Cox proportional hazards regression model was utilized to elucidate the non-linear relationship between HDL-C levels and the reversion to normoglycemia from Pre-DM. Subsequently, a log-likelihood ratio test was conducted to determine the optimal model for elucidating the correlation between the abovementioned variables.

Employing a stratified Cox proportional hazard regression model, subgroup analyses were undertaken across diverse categories encompassing the family history of diabetes, DBP, age, sex, drinking status, SBP, BMI, and smoking status. Initially, continuous data, including SBP and age, were discretized into categorical variables using clinical thresholds (age: < 30, 30 to 60, ≥ 60 years; DBP: < 90, ≥ 90 mmHg)^29^. In addition to accounting for the stratification factor, adjustments were made for various covariates, including DBP, age, sex, SBP, BMI, FPG, AST, BUN, ALT, LDL-c, Scr, TG, family history of diabetes, drinking status, and smoking status. The presence of interaction terms in models with and without such terms was then determined using the likelihood ratio test. The STROBE statement was adhered to for reporting all findings^30^. Statistical significance was determined using a two-tailed test with a threshold of P < 0.05.

### Ethics approval and consent to participate

The original study followed guidelines outlined by the Helsinki Declaration and was approved by the Rich Healthcare Group Review Board. In addition, the Rich Healthcare Group Review Board has waived informed consent for the current retrospective study. All methods were performed in accordance with the relevant the Declaration of Helsinki.

## Results

### Characteristics of participants

Table 1 presented the medical characteristics and demographic of the research participants. The mean age was 50.921 ± 13.437 years, with 5414 (35.110%) female participants. The median follow-up time was 2.883 years, and 6627 (42.977%) participants experienced a final reversion to normoglycemia from Pre-DM. The mean ± SD HDL-C level was 1.335 ± 0.303 mmol/L. We categorized adults into four groups based on HDL-C quartiles (Q1 ≤ 1.120, 1.120 < Q2 ≤ 1.310, 1.310 < Q3 ≤ 1.510, Q4 > 1.510). Age, BMI, DBP, TG, AST, ALT, and Scr were significantly higher in the three lower HDL-C groups compared to the highest HDL-C group (Q4). In contrast to the other three groups, the highest HDL-C group (Q4) had the highest levels of LDL-C and TC. Additionally, the lowest HDL-C group (Q1) had a higher percentage of males, current smokers, and current drinkers.

**Table 1:** The baseline characteristics of participants.

HDL-C (mmol/L)	All participants	Q1(≤ 1.120)	Q2(1.120 to ≤ 1.310)	Q3(1.310 to ≤ 1.510)	Q4(> 1.510)	P-value
Participants	15,420	3795	3795	3860	3970	
Gender						< 0.001
Male	10,006 (64.890%)	2966 (78.155%)	2645 (69.697%)	2380 (61.658%)	2015 (50.756%)	
Female	5414 (35.110%)	829 (21.845%)	1150 (30.303%)	1480 (38.342%)	1955 (49.244%)	
Age (years)	50.921 ± 13.437	50.932 ± 13.050	50.351 ± 13.586	50.267 ± 13.505	52.093 ± 13.519	< 0.001
Smoking status						< 0.001
Current-smoker	3414 (22.140%)	1108 (29.196%)	894 (23.557%)	781 (20.233%)	631 (15.894%)	
Ex-smoker	650 (4.215%)	181 (4.769%)	180 (4.743%)	153 (3.964%)	136 (3.426%)	
Never-smoker	11,356 (73.645%)	2506 (66.034%)	2721 (71.700%)	2926 (75.803%)	3203 (80.680%)	
Drinking status						0.002
Current-drinker	630 (4.086%)	160 (4.216%)	145 (3.821%)	149 (3.860%)	176 (4.433%)	
Ex-drinker	2642 (17.134%)	657 (17.312%)	722 (19.025%)	658 (17.047%)	605 (15.239%)	
Never-drinker	12,148 (78.781%)	2978 (78.472%)	2928 (77.154%)	3053 (79.093%)	3189 (80.327%)	
Family history of diabetes						0.230
No	15,020 (97.406%)	3684 (97.075%)	3690 (97.233%)	3765 (97.539%)	3881 (97.758%)	
Yes	400 (2.594%)	111 (2.925%)	105 (2.767%)	95 (2.461%)	89 (2.242%)	
SBP (mmHg)	127.500 ± 17.696	127.700 ± 16.948	127.516 ± 17.209	127.353 ± 17.835	127.437 ± 18.696	0.848
DBP (mmHg)	78.504 ± 11.192	79.364 ± 10.826	78.451 ± 11.108	78.351 ± 11.318	77.880 ± 11.443	< 0.001
BMI (kg/m^2^)	24.834 ± 3.317	25.785 ± 3.136	25.074 ± 3.237	24.662 ± 3.232	23.862 ± 3.359	< 0.001
AST (U/L)	26.179 ± 11.863	27.325 ± 11.767	26.019 ± 13.119	25.935 ± 11.374	25.473 ± 11.055	< 0.001
ALT (U/L)	22.000 (15.500–33.000)	25.800 (18.000–38.450)	22.300 (16.000–33.000)	21.000 (15.000–31.625)	19.200 (14.000–28.675)	< 0.001
HDL-C (mmol/L)	1.335 ± 0.303	0.978 ± 0.115	1.222 ± 0.055	1.408 ± 0.058	1.715 ± 0.232	< 0.001
TG (mmol/L)	1.450 (1.000–2.160)	1.910 (1.330–2.800)	1.500 (1.030–2.120)	1.330 (0.940–1.910	1.170 (0.820–1.710)	< 0.001
LDL-C (mmol/L)	2.931 ± 0.722	2.833 ± 0.778	2.811 ± 0.701	2.949 ± 0.660	3.123 ± 0.701	< 0.001
TC (mmol/L)	5.044 ± 0.948	4.853 ± 0.952	4.828 ± 0.949	5.038 ± 0.856	5.436 ± 0.905	< 0.001
BUN (mmol/L)	5.005 ± 1.242	4.956 ± 1.204	5.003 ± 1.254	5.024 ± 1.238	5.037 ± 1.268	0.025
Scr (umol/L)	72.991 ± 16.173	74.821 ± 15.724	74.323 ± 16.038	73.224 ± 16.393	69.742 ± 16.034	< 0.001
FPG (mmol/L)	5.953 ± 0.320	5.979 ± 0.325	5.951 ± 0.315	5.944 ± 0.319	5.941 ± 0.317	< 0.001

Values are n (%) or mean ± SD or median (quartile).

*HDL-C* high-density lipoprotein cholesterol, *SBP* systolic blood pressures, *DBP* diastolic blood pressures, *BMI* body mass index, *AST* aspartate aminotransferase, *ALT* alanine aminotransferase, *LDL-C* low-density lipoprotein cholesterol, *TC* total cholesterol, *TG* triglycerides, *Scr* serum creatinine, *BUN* blood urea nitrogen, *FPG* fasting plasma glucose.

### The reversal rate to normoglycemia from Pre-DM

After a median follow-up time of 2.883 years, 6627 (42.977%) patients with Pre-DM had the reversion to normoglycemia. The rate of glycemic reversal in patients with Pre-DM was: overall group: 14.538, Q1 group: 12.228, Q2 group: 13.861, Q3 group: 15.606, and Q4 group: 16.750 per 100 person-years, respectively. During a median follow-up period of 2.883 years, the cumulative reversal rates of Pre-DM to normoglycemia were: overall group: 42.977% (42.195–43.758), Q1 group: 39.499% (37.943–41.055%), Q2 group: 41.555% (39.986–43.123%), Q3: 44.275% (42.707–45.842%), and Q4 group 46.398% (44.846–47.950%) (Table 2). Significantly more participants with higher HDL-C levels had a reversal rate than those with lower HDL-C levels (P < 0.001 for trend).

**Table 2:** The rate of reversion from Pre-DM to normoglycemia.

HDL-C	Participants (n)	Reversion events (n)	Cumulative incidence (95% CI) (%)	Per 100 person-year
Overall	15,420	6627	42.977 (42.195–43.758)	14.538
Q1	3795	1499	39.499 (37.943–41.055)	12.228
Q2	3795	1577	41.555 (39.986–43.123)	13.861
Q3	3860	1709	44.275 (42.707–45.842)	15.606
Q4	3970	1842	46.398 (44.846–47.950)	16.750
P for trend			< 0.001	< 0.001

*HDL-C* high-density lipoprotein cholesterol, *CI* confidence interval.

### Factors influencing reversion from Pre-DM to normoglycemia

Univariate analyses revealed significant negative correlations between reversion from Pre-DM to normoglycemia and factors including AST, age, TG, DBP, SBP, ALT, LDL-C, BMI, BUN, TC, and family history of diabetes. Conversely, a positive relationship was observed between reversion from Pre-DM to normoglycemia and factors including never drinking, HDL-C, and never smoking, as shown in Table 3.

**Table 3:** The results of the univariate Cox proportional hazards regression.

	Statistics	HR (95%CI)	P value
Gender			< 0.001
Male	10,006 (64.890%)	ref	
Female	5414 (35.110%)	1.251 (1.190, 1.314)	
Age(years)	50.921 ± 13.437	0.973 (0.971, 0.975)	< 0.001
Drinking status			
Current-drinker	630 (4.086%)	ref	
Ex-drinker	2642 (17.134%)	1.055 (0.918, 1.211)	0.451
Never- drinker	12,148 (78.781%)	1.148 (1.010, 1.306)	0.035
Smoking status			
Current-smoker	3414 (22.140%)	ref	
Ex-smoker	650 (4.215%)	1.065 (0.931, 1.218)	0.361
Never-smoker	11,356 (73.645%)	1.262 (1.187, 1.342)	< 0.001
Family history of diabetes			0.010
No	15,020 (97.406%)	ref	
Yes	400 (2.594%)	0.818 (0.702, 0.954)	
SBP (mmHg)	127.500 ± 17.696	0.988 (0.987, 0.990)	< 0.001
DBP (mmHg)	78.504 ± 11.192	0.984 (0.982, 0.986)	< 0.001
BMI (kg/m^2^)	24.834 ± 3.317	0.933 (0.925, 0.940)	< 0.001
AST (U/L)	26.179 ± 11.863	0.987 (0.985, 0.990)	< 0.001
ALT (U/L)	28.156 ± 23.145	0.994 (0.992, 0.995)	< 0.001
HDL-C (mmol/L)	1.335 ± 0.303	1.920 (1.798, 2.050)	< 0.001
TG (mmol/L)	1.806 ± 1.410	0.900 (0.881, 0.919)	< 0.001
LDL-C (mmol/L)	2.931 ± 0.722	0.935 (0.904, 0.967)	< 0.001
TC (mmol/L)	5.044 ± 0.948	0.884 (0.861, 0.907)	< 0.001
BUN (mmol/L)	5.005 ± 1.242	0.959 (0.940, 0.978)	< 0.001
Scr (umol/L)	72.991 ± 16.173	0.997 (0.995, 0.998)	< 0.001
FPG (mmol/L)	5.953 ± 0.320	0.209 (0.189, 0.231)	< 0.001

*HDL-C* high-density lipoprotein cholesterol, *SBP* systolic blood pressures, *DBP* diastolic blood pressures, *BMI* body mass index, *AST* aspartate aminotransferase, *ALT* alanine aminotransferase, *LDL-C* low-density lipoprotein cholesterol, *TC* total cholesterol, *TG* triglycerides, *Scr* serum creatinine, *BUN* blood urea nitrogen, *FPG* fasting plasma glucose, *HR* hazard ratios, *CI* confidence interval.

**Figure 2:**
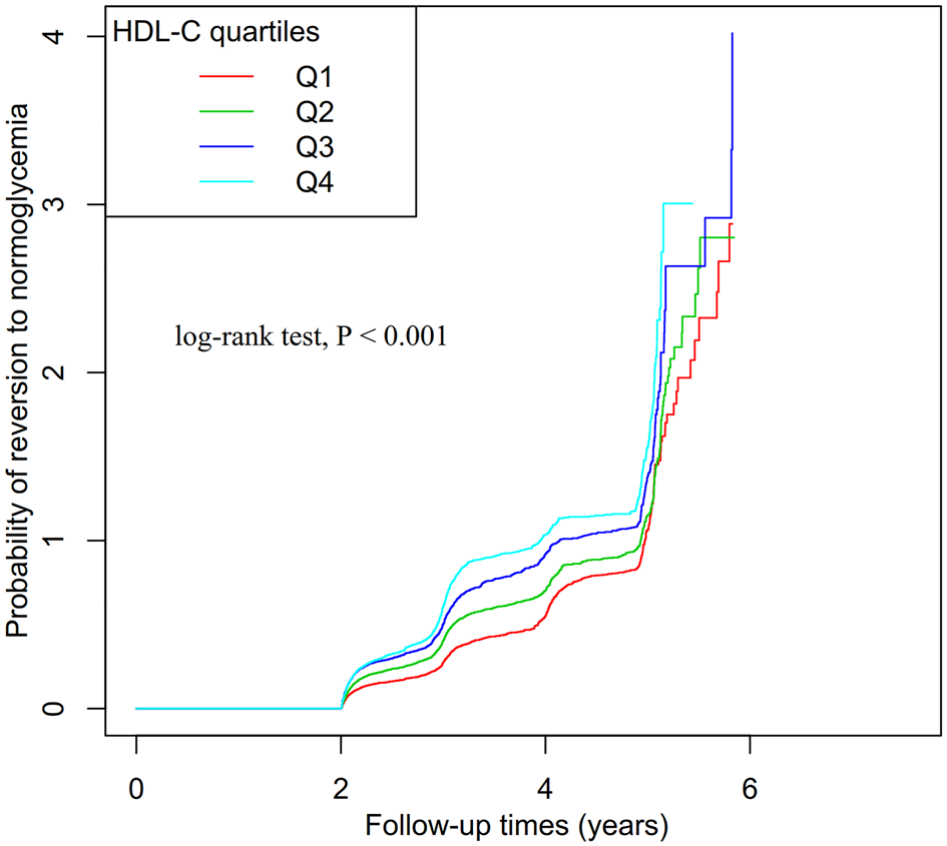
Kaplan–Meier event-free survival curve. Kaplan–Meier event-free survival curve. Kaplan–Meier analysis of incident reversion to normoglycemia from Pre-DM based on HDL-C levels quartiles (log-rank, P < 0.001).

Figure 2 illustrates the Kaplan–Meier curves depicting the likelihood of the reversion from Pre-DM to normoglycemia, categorized by quartiles of HDL-C. The probability of transitioning back to normoglycemia from Pre-DM significantly differed across the HDL-C groups (log-rank test, P < 0.001). Moreover, the probability of reversion from Pre-DM to normoglycemia gradually increased with increasing levels of HDL-C. This implies that individuals with the highest HDL-C levels possessed the greatest likelihood of reversion from Pre-DM to normoglycemia.

### The connection between HDL-C levels and reversion from Pre-DM to normoglycemia

We employed the multivariate Cox proportional hazards regression model to construct three models to examine the connection between HDL-C levels and the reversion from Pre-DM to normoglycemia. In Model 1, a 1 mmol/L rise in HDL-C levels was associated with a 92.0% increase in the likelihood of the reversion from Pre-DM to normoglycemia (HR 1.920, 95% CI 1.798–2.050, P < 0.001). In Model 2, after adjusting for family history of diabetes, DBP, age, sex, drinking status, SBP, BMI, and smoking status, each 1 mmol/L increase in HDL-C levels was related to an 88.3% higher probability of reverting to normoglycemia (HR 1.883, 95% CI 1.752–2.023, P < 0.001). In Model 3, after adjusting for various factors, including DBP, smoking status, SBP, BMI, gender, age, drinking status, FPG, AST, BUN, ALT, LDL-C, Scr, TG, and family history of diabetes, the HR between HDL-C levels and reversion to normoglycemia from Pre-DM was found to be 1.898 (95% CI: 1.758–2.048, P < 0.001). The confidence intervals in Table 4 indicate that the connection between HDL-C levels and reversion from Pre-DM to normoglycemia, as determined by the model, is statistically reliable.

**Table 4:** Relationship between HDL-C levels and reversion to normoglycemia from Pre-DM in different models.

Exposure	Model 1 (HR., 95% CI, P)	Model 2 (HR, 95% CI, P)	Model 3 (HR, 95% CI, P)	Model 4 (HR, 95% CI, P)
HDL-C	1.920 (1.798, 2.050) < 0.001	1.883 (1.752, 2.023) < 0.001	1.898 (1.758, 2.048) < 0.001	1.948 (1.804, 2.102) < 0.001
HDL-C (quartiles)				
Q1	ref	ref	ref	ref
Q2	1.244 (1.159, 1.335) < 0.001	1.212 (1.129, 1.301) < 0.001	1.198 (1.115, 1.288) < 0.001	1.204 (1.120, 1.294) < 0.001
Q3	1.569 (1.463, 1.682) < 0.001	1.487 (1.385, 1.597) < 0.001	1.470 (1.368, 1.580) < 0.001	1.491 (1.385, 1.604) < 0.001
Q4	1.794 (1.675, 1.922) < 0.001	1.742 (1.620, 1.873) < 0.001	1.733 (1.609, 1.866) < 0.001	1.767 (1.637, 1.908) < 0.001
P for trend	< 0.001	< 0.001	< 0.001	< 0.001

Model 1: we did not adjust for other covariants.

Model 2: we adjusted for DBP, SBP, BMI, gender, age, drinking status, family history of diabetes, and smoking status.

Model 3: we adjusted for DBP, SBP, BMI, gender, age, drinking status, family history of diabetes, smoking status, FPG, AST, BUN, ALT, LDL-C, Scr, and TG.

Model 4: we adjusted for DBP (smooth), age (smooth), sex, SBP (smooth), BMI (smooth), drinking status, family history of diabetes, smoking status, FPG (smooth), AST (smooth), BUN(smooth), ALT(smooth), LDL-C (smooth), Scr (smooth), and TG (smooth).

*HR* hazard ratios, *CI* confidence interval, *Ref* reference, *GAM* generalized additive mode, *HDL-C* high-density lipoprotein cholesterol.

Furthermore, we discretized the HDL-C levels from a continuous variable to a categorical variable and subsequently reintegrated the categorically transformed HDL-C levels into the model. In model 3, the HR was 1.198 (95% CI 1.115–1.288) for the Q2 group, 1.470 (95% CI 1.368–1.580) for the Q3 group, and 1.733 (95% CI 1.609–1.866) for the Q4 group compared with subjects in the Q1 group.

### Sensitivity analysis

A series of sensitivity analyses were conducted to ensure the robustness of our findings. Initially, we incorporated the continuity covariate as a curve into the equation using a GAM. As indicated in Table 4, the results of Model 4 were consistent with the fully adjusted model (Model 3). Specifically, subjects in the group with the highest HDL-C levels (Q4) were 76.7% more likely to return to normoglycemia from Pre-DM compared to those in the group with the lowest HDL-C levels (Q1) (HR 1.767, 95% CI 1.637–1.908, P < 0.001).

Additionally, a sensitivity analysis was conducted on participants with a BMI less than 25 kg/m^2^. Following the adjustment for various confounding variables, such as DBP, age, sex, SBP, BMI, FPG, AST, BUN, ALT, LDL-C, Scr, TG, family history of diabetes, drinking status, and smoking status, the findings revealed a positive correlation between HDL-C levels and the reversion to normoglycemia from Pre-DM (HR 1.831, 95% CI 1.665–2.012, P < 0.001). Furthermore, patients aged 60 years or older were excluded from the analysis. After accounting for confounding variables, the results continued to demonstrate a positive association between HDL-C levels and the reversion to normoglycemia (HR 1.997, 95% CI: 1.840–2.167, P < 0.001) (Table 5). Based on the comprehensive sensitivity analyses conducted, it becomes apparent that our findings possess a high degree of robustness. Furthermore, in order to assess the susceptibility to unmeasured confounding, we employed the calculation of an E-value. The results indicate that the influence of unknown or unmeasured variables on the correlation between HDL-C levels and the transition from Pre-DM to normoglycemia is minimal, as the E-value (2.49) surpasses the relative risk associated with HDL-C levels and unmeasured confounders (1.87).

**Table 5:** Relationship between HDL-C levels and reversion to normoglycemia from Pre-DM in different sensitivity analyses.

Exposure	Model 5 (HR, 95%CI, P)	Model 6 (HR, 95%CI, P)
HDL-C	1.831 (1.665, 2.012) < 0.001	1.997 (1.840, 2.167) < 0.001
HDL-C (quartiles)		
Q1	ref	ref
Q2	1.149 (1.040, 1.270) 0.006	1.220 (1.126, 1.323) < 0.001
Q3	1.397 (1.266, 1.541) < 0.001	1.525 (1.407, 1.653) < 0.001
Q4	1.716 (1.555, 1.894) < 0.001	1.835 (1.687, 1.995) < 0.001
P for trend	< 0.001	< 0.001

Model 5 was sensitivity analysis in participants without BMI ≥ 25 kg/m2. We adjusted DBP, SBP, BMI, gender, age, drinking status, family history of diabetes, smoking status, FPG, AST, BUN, ALT, LDL-C, Scr, and TG.

Model 6 was sensitivity analysis in participants without age ≥ 60 years. We adjusted DBP, SBP, BMI, gender, age, drinking status, family history of diabetes, smoking status, FPG, AST, BUN, ALT, LDL-C, Scr, and TG.

*HR* hazard ratios, *CI* confidence, *Ref* reference, *HDL-C* high-density lipoprotein cholesterol.

### The analyses of non-linear relationship between HDL-C levels and reversion from Pre-DM to normoglycemia

**Figure 3:**
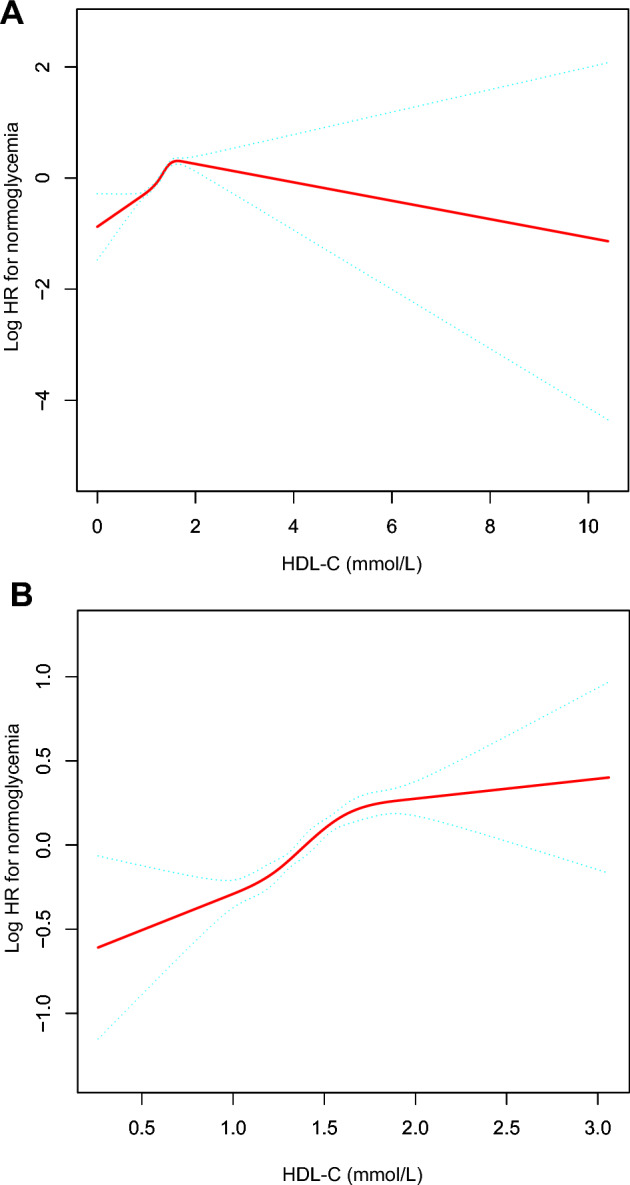
(**A**) The relationship between HDL-C levels and the reversion to normoglycemia from Pre-DM stratified by gender. A non-linear relationship between HDL-C levels and reversion to normoglycemia from Pre-DM was detected in males after adjustment for DBP, SBP, BMI, age, drinking status, family history of diabetes, smoking status, FPG, AST, BUN, ALT, LDL-C, Scr, and TG. (**B**) The relationship between HDL-C levels and the reversion to normoglycemia from Pre-DM stratified by gender. A non-linear relationship between HDL-C levels and reversion to normoglycemia from Pre-DM was detected in females after adjustment for DBP, SBP, BMI, age, drinking status, family history of diabetes, smoking status, FPG, AST, BUN, ALT, LDL-C, Scr, and TG.

Utilizing a Cox proportional hazards regression model with cubic spline functions and smooth curve fitting, our analysis revealed a nonlinear association between HDL-C levels and the likelihood of reverting to normoglycemia in individuals with Pre-DM stratified by gender (Fig. 3A and Fig. 3B). Furthermore, employing a standard binary two-piecewise Cox proportional-hazards regression model to assess the data, we employed the log-likelihood ratio test to determine the most suitable model fit (Table 6). Employing the recursive technique, we identified the HDL-C inflection point as 1.540 mmol/L in males and 1.620 mmol/L in females. There was a strong positive correlation between HDL-C and the reversion from Pre-DM to normoglycemia on the left of the inflection point (Male: HR 2.783, 95% CI 2.373–3.263; Female: HR 2.217, 95% CI 1.802–2.727). However, on the right side of the inflection point, the relationship between HDL-C and the reversion from Pre-DM to normoglycemia was not significant (Male: HR 0.926, 95% CI 0.666–1.288; Female: HR 1.266, 95% CI 0.906–1.770) (Table 6).

**Table 6:** The result of the two-piecewise Cox proportional hazards regression model.

Outcome: reversion to normoglycemia	All participants (HR, 95% CI, P)	Male	Female
Standard Cox regression	1.898 (1.758, 2.048) < 0.001	1.923 (1.753, 2.110) < 0.001	1.832 (1.597, 2.101) < 0.001
Fitting model by two-piecewise Cox proportional hazards regression			
The inflection point of HDL-C	1.560	1.540	1.620
≤ inflection point	2.599 (2.289, 2.950) < 0.001	2.783 (2.373, 3.263) < 0.001	2.217 (1.802, 2.727) < 0.001
> inflection point	1.103 (0.889, 1.368) 0.373	0.926 (0.666, 1.288) 0.648	1.266 (0.906, 1.770) 0.167
P for the log-likelihood ratio test	< 0.001	< 0.001	0.015

Note 1: In all participants, we adjusted DBP, SBP, BMI, gender, age, drinking status, family history of diabetes, smoking status, FPG, AST, BUN, ALT, LDL-C, Scr, and TG.

Note 2: For Male and Female subgroups, we adjusted for DBP, SBP, BMI, age, drinking status, family history of diabetes, smoking status, FPG, AST, BUN, ALT, LDL-C, Scr, and TG.

*HR* hazard ratios, *CI* confidence, *HDL-C* high-density lipoprotein cholesterol.

### The results of subgroup analyses

In all predetermined or exploratory subgroups analyzed (Table 7), the association between HDL-C levels and reversion to normoglycemia from Pre-DM was not influenced by age, sex, DBP, drinking status, or family history of diabetes (P > 0.05 for interaction). However, among patients with SBP < 140 mmHg and those who were ever smokers, there was a more pronounced correlation between HDL-C levels and reversion to normoglycemia from Pre-DM. Conversely, a weaker correlation was observed among never smokers, current smokers, and individuals with SBP ≥ 140 mmHg.

**Table 7:** Effect size of HDL-C levels and reversion to normoglycemia from Pre-DM in prespecified and exploratory subgroups.

Characteristic	No of participants	HR (95% CI) P value	P for interaction
Age (years)			0.1007
< 30	593	2.638 (1.841, 3.780) < 0.001	
30–60	10,495	1.836 (1.686, 1.999) < 0.001	
≥ 60	4332	1.708 (1.434, 2.035) < 0.001	
Gender			0.4297
Male	10,006	1.936 (1.771, 2.115) < 0.001	
Female	5414	1.820 (1.600, 2.070) < 0.001	
BMI (kg/m^2^)			0.0928
< 25	8233	1.845 (1.688, 2.017) < 0.001	
≥ 25	7187	2.121 (1.842, 2.444) < 0.001	
SBP (mmHg)			0.0016
< 140	11,963	1.995 (1.843, 2.159) < 0.001	
≥ 140	3457	1.475 (1.233, 1.764) < 0.001	
DBP (mmHg)			0.0642
< 90	13,128	1.947 (1.798, 2.108) < 0.001	
≥ 90	2292	1.593 (1.297, 1.957) < 0.001	
Smoking status			0.0003
Current smoker	3414	2.176 (1.804, 2.624) < 0.001	
Ever smoker	650	4.321 (2.759, 6.766) < 0.001	
Never smoker	11,356	1.806 (1.656, 1.969) < 0.001	
Drinking status			0.3488
Current drinker	630	2.396 (1.570, 3.657) < 0.001	
Ever drinker	2642	2.062 (1.692, 2.514) < 0.001	
Never drinker	12,148	1.861 (1.712, 2.022) < 0.001	
Family history of diabetes			0.7383
No	15,020	1.894 (1.754, 2.046) < 0.001	
Yes	400	2.085 (1.194, 3.643) 0.010	

Note 1: Above model was adjusted for DBP, SBP, BMI, gender, age, drinking status, family history of diabetes, smoking status, FPG, AST, BUN, ALT, LDL-C, Scr, and TG.

Note 2: The model is not adjusted for the stratification variable in each case.

## Discussion

HDL-C levels and the reversion to normoglycemia from Pre-DM. Our findings indicate a significant positive connection between the increase in HDL-C levels and the probability of regression from Pre-DM to normoglycemia. Furthermore, a threshold effect curve was observed, revealing the distinct connection between HDL-C levels and the reversion to normoglycemia from Pre-DM on either side of the inflection point. Additionally, our study identified that individuals with SBP < 140 mmHg and ever smoker exhibited a stronger correlation between HDL-C levels and the reversion to normoglycemia from Pre-DM.

A prospective cohort investigation involving 491 subjects showed that 22.6% of those with Pre-DM experienced a return to normoglycemia over a median follow-up period of 2.5 years^31^. In addition, the findings of another study after 1 year of follow-up showed that 54% of subjects with Pre-DM returned to normal blood glucose, while 6% developed DM^15^. Furthermore, a cohort study in China, encompassing 14,231 Chinese adults, revealed that 44.9% of patients with Pre-DM reverted to normoglycemia within two years^32^. Our investigation revealed that 6,627 patients with Pre-DM achieved normoglycemia after a median follow-up duration of 2 years. Differences in the rate of reversion to normoglycemia from Pre-DM across various studies can be ascribed to variations in participants' age, duration of follow-up, and ethnic background. It is crucial to acknowledge that all studies have consistently confirmed a significant proportion of individuals with Pre-DM experiencing a return to normoglycemia. Consequently, identifying the factors that contribute to this reversion is of utmost importance in diabetes prevention and the mitigation of associated complications.

Numerous previous studies have indicated a correlation between diminished HDL-C levels and elevated susceptibility to diabetes^33,34^. Furthermore, HDL-C levels in prediabetic subjects were negatively connected to the likelihood of developing diabetes^17,18^. Moreover, lower HDL-C levels have been linked to a substantial risk factor for Pre-DM^35,36^. Consequently, we postulate that a decline in HDL-C levels might be linked to an augmented probability of transitioning from prediabetes to normoglycemia. Regrettably, there is a lack of literature exploring the interrelation between them. In a longitudinal study comprising 1329 individuals diagnosed with prediabetes, the findings of a Cox proportional hazards multivariate model indicated that a 1 mmol/L elevation in HDL-C levels was linked to a 70% increase in the likelihood of achieving normoglycemia among subjects with Pre-DM (HR: 1.70, 95% CI: 1.23–2.34)^21^. Another investigation involving 817 individuals diagnosed with prediabetes from Germany has demonstrated that for every 10 mg/dL increase in HDL-C levels, there was a 17% higher probability of attaining normoglycemia in patients with prediabetes after controlling for residential traffic intensity, mental distress, age, lifestyle factors, educational level, anthropometric markers, and sex (HR 1.17, 95% CI 1.02–1.35)^20^. Our study served as a valuable addition to the current body of literature, as it provided further support for the hypothesis that a decrease in HDL-C levels is linked to a diminished likelihood of the reversion from Pre-DM to normoglycemia. In contrast to previous studies, our research employed both categorical and continuous variables of HDL-C levels as independent variables, enabling a more comprehensive exploration of their relationship with the reversion from prediabetes to normoglycemia. This approach minimized the loss of pertinent information and facilitated the quantification of their association. Furthermore, our study employed distinct covariates for adjustment compared to previous studies. We accounted for more parameters: ALT, smoking status, Scr, LDL-C, AST, FPG, drinking status, and BUN^37–41^. Extensive evidence has demonstrated the association between these parameters and the onset of diabetes. Additionally, our sensitivity analysis revealed that this association persists among individuals with a BMI < 25 kg/m^2^ and age < 60 years. The results have substantiated the enduring correlation between HDL-C levels and the reversion from Pre-DM to normoglycemia. This discovery offers a valuable point of reference for clinical interventions targeting HDL-C levels to augment the likelihood of the reversion from Pre-DM to normoglycemia. Notably, this study, which investigates nonlinearity, represents a significant advancement compared to prior research endeavors.

The precise mechanism for connecting HDL-C levels and the reversion from Pre-DM to normoglycemia is not yet fully understood. The following mechanisms may explain the ability of HDL-C to contribute to reversion from Pre-DM to normoglycemia. Prior research has demonstrated that HDL-C possesses antioxidant and anti-inflammatory properties^42^. By preventing the apoptosis that is triggered by inflammatory cytokines or oxidized low-density lipoprotein, such as tumor necrosis factor-alpha, HDL-C can directly increase the viability of islet cells^43^. Additionally, by triggering the Adenosine 5'-monophosphate-activated protein kinase pathway, HDL-C increases skeletal muscle cells' sensitivity to insulin and glucose absorption^44^. Furthermore, HDL-C promotes insulin secretion by encouraging the outflow of cholesterol from pancreatic β cells^45^. These mechanisms might logically explain the elevation in HDL-C associated with a greater chance of the reversion from Pre-DM to normoglycemia.

Moreover, the present study employed a two-piecewise Cox proportional hazards regression model to elucidate the nonlinear relationships. The results revealed a nonlinear association and a threshold effect between HDL-C levels and the reversion from Pre-DM to normoglycemia. After accounting for confounding factors, the inflection point of HDL-C levels was determined to be 1.540 mmol/L in males and 1.620 mmol/L in females. Notably, no significant correlation was observed between elevated HDL-C levels and the reversion to normoglycemia from Pre-DM when HDL-C levels exceeded the inflection point. However, when the HDL-C levels were below the inflection point, the likelihood of returning to normoglycemia increased significantly by 1.783-fold in males and 1.217-fold in females for every 1 mmol/L increase in HDL-C levels. This suggests that the probability of the reversion from Pre-DM to normoglycemia gradually rises with increasing HDL-C levels in individuals with Pre-DM. However, once the HDL-C level reaches an inflection point, the probability of reversion to normoglycemia from Pre-DM stabilizes rather than continues to increase. The nonlinear relationship between HDL-C and the reversion to normoglycemia from prediabetes might be due to the influence of other covariates at baseline. Compared to those on the left side of the inflection point, participants on the right side tend to be older, with higher TC, LDL-C, and BUN levels and a higher proportion of current drinkers (Table S2A and Table S2B). Previous studies have indicated that these covariates inhibit the reversion of normoglycemia from prediabetes^18,21,46^. Therefore, we speculate that once HDL-C exceeds the inflection point, its role in promoting the reversion to normoglycemia from prediabetes might be weakened due to the influence of age, TC, LDL-C, BUN, and alcohol consumption. This discovery of a curvilinear association between HDL-C and the reversion to normoglycemia from Pre-DM holds significant clinical value, as it benefits clinical advice and provides a useful reference for decision-making processes to optimize preventive DM strategies. Individuals with pre-DM are not only at risk for the development of T2DM but also for the onset of all-cause mortality and cardiovascular disease^47,48^. Prior studies have indicated that even a brief restoration to normoglycemia is linked to a substantially reduced risk of T2DM development in individuals with Pre-DM^12^. Consequently, the treatment of Pre-DM should prioritize achieving a return to normoglycemia rather than focusing solely on mitigating the potential consequences of Pre-DM and reducing the possibility of developing T2DM. Our work established an HDL-C threshold for the return to normoglycemia in Chinese people with pre-DM. In other words, keeping HDL-C levels near the inflection point in patients with prediabetes may greatly increase the likelihood of reversion from pre-DM to normoglycemia.

Based on previous studies, there are methods to increase HDL-C levels. Lifestyle modifications, including regular physical activity, weight management, smoking cessation, and dietary changes, are fundamental in the augmentation of HDL-C concentrations^49,50^ and can also increase the likelihood of reversion from pre-DM to normoglycemia^9,51^. In addition, some drugs also increased HDL-C levels, but they remained controversial for improving insulin resistance and glucose metabolism. Niacin has been used to increase HDL-C levels and improve lipid profiles significantly, but it may also worsen insulin resistance^52^. Statins, while primarily used to lower LDL-C, can have a modest effect on raising HDL-C levels. Some studies suggest that statins may increase insulin resistance^53,54^. Cholesteryl ester transfer protein inhibitors are of interest to researchers because they not only have the potential to increase HDL-C levels but also have been associated with a reduction in the incidence of diabetes and an improvement in glucose metabolism^55^.

Subgroup analyses showed that individuals with SBP < 140 mmHg and ever-smokers exhibited a stronger correlation between HDL-C levels and the reversion to normoglycemia from Pre-DM. We observed that participants with SBP < 140 mmHg exhibited a lower BMI (Table S3A). Previous studies have suggested that lower SBP and a reduced BMI may indicate diminished insulin resistance^56,57^. In contrast, ever-smokers exhibited higher BMI (Table S3B). A review of the existing literature indicated that an increase typically followed the cessation of smoking in body weight^58^. Although smoking cessation might lead to weight gain, it also improves HDL-C, total high-density lipoprotein, and the size of high-density lipoprotein particles, with these positive changes in HDL-C mitigating the negative impact of weight gain^58^. Moreover, smoking cessation has enhanced high-density lipoprotein functionality, boosting cholesterol efflux capacity and reducing inflammation without altering HDL-C levels or high-density lipoprotein subtypes^59^. We postulated that HDL-C levels and the reversion to normoglycemia from prediabetes were more pronounced in participants with SBP < 140 mmHg and ever-smokers due to the above reasons.

This research possesses several noteworthy strengths. Firstly, it uncovers the non-linear relationship between HDL-C and the reversion from Pre-DM to normoglycemia and identifies crucial inflection points. Secondly, a multiple imputation approach was employed to address the issue of missing data, enabling the attainment of optimal statistical power while mitigating bias arising from absent covariate information. Lastly, a set of sensitivity analyses were performed to ascertain the results' dependability.

The study has several potential limitations that should be acknowledged. Firstly, the homogeneity of the participant sample, consisting solely of individuals of Chinese descent, necessitates further investigation to establish the relationship between HDL-C and return to normoglycemia in individuals with Pre-DM from diverse genetic backgrounds. Secondly, it is important to note that relying solely on FPG measurements may not fully capture the complexity of prediabetes. However, these additional assessments were not feasible due to logistical challenges associated with measuring HbA1c levels and 2-h oral glucose tolerance tests within a large study cohort. Future research endeavors will address this limitation by conducting our own study or collaborating with other researchers to gather data on 2-h oral glucose tolerance tests and HbA1c levels. Furthermore, it should be noted that this study relies on a secondary analysis of previously published data, which limits our ability to control for variables that were not originally included in the dataset, such as insulin concentration and the use of lipid-lowering medications. Nevertheless, we employed the E-value to quantify the potential influence of unmeasured confounders and determined that these confounders are unlikely to account for the observed outcomes. Additionally, this is a secondary retrospective study, and the data was downloaded from the DATADRYAD database. Only baseline HDL-C was measured in the previous original study, and the original research did not involve changes in HDL-C over time. Because our study was observational, we cannot determine a causal relationship between HDL-C and the reversion from prediabetes to normoglycemia. In the future, we can consider designing our studies to collect as many variables as possible, including information on the evolution of HDL-C during follow-up. Therefore, we can observe the changes in HDL-C and further explore the effect of changes in HDL-C on the reversion from pre-DM to normoglycemia in the future through a generalized additive mixed model.

## Conclusion

This study demonstrated an independent connection between HDL-C and regression to normoglycemia in Chinese adults with Pre-DM, revealing a specific non-linear relationship and threshold effect. A significant positive connection was observed between HDL-C levels and the regression from Pre-DM to normoglycemia to normoglycemia, particularly when HDL-C levels were below the inflection point. Consequently, keeping HDL-C levels near the inflection point in patients with prediabetes may greatly increase the likelihood of reversion from pre-DM to normoglycemia.

### Supplementary Information

Supplementary Tables.

## Data Availability

The data are available from the ‘DataDryad’ database (https://datadryad.org/stash/dataset/doi:10.5061%2Fdryad.ft8750v).

## Abbreviations

DM: Diabetes mellitus
T2DM: Type 2 diabetes mellitus
Pre-DM: Prediabetes
ALT: Alanine aminotransferase
AST: Aspartate aminotransferase
BMI: Body mass index
SBP: Systolic blood pressure
DBP: Diastolic blood pressure
FPG: Fasting plasma glucose
TC: Total cholesterol
TG: Triglyceride
HDL-C: High-density lipoprotein cholesterol
LDL-C: Low-density lipid cholesterol
BUN: Blood urea nitrogen
Scr: Serum creatinine
MAR: Missing-at-random
GAM: Generalized additive models
HR: Hazard ratios
CI: Confidence intervals
Ref: Reference
