# Supplementary material for: Association between high-density lipoprotein cholesterol and reversion to normoglycemia from prediabetes: an analysis based on data from a retrospective cohort study
Source: Sci Rep. 2024 Jan 2;14:35. doi: 10.1038/s41598-023-50539-w (PMC10762102; doi:10.1038/s41598-023-50539-w)
Supplement: Supplementary file 1 — Supplementary Tables. [file 41598_2023_50539_MOESM1_ESM.docx]

**Table S1** The results of the collinearity screening

|  | Step 1 | Step 2 |
| --- | --- | --- |
| Gender | 2.1 | 2.1 |
| Age(years) | 1.4 | 1.4 |
| Drinking status | 1.1 | 1.1 |
| Smoking status | 1.3 | 1.3 |
| Family history of diabetes | 1 | 1 |
| SBP (mmHg) | 1.9 | 1.9 |
| DBP (mmHg) | 1.8 | 1.8 |
| BMI (kg/m^2^) | 1.3 | 1.3 |
| AST (U/L) | 3.2 | 3.2 |
| ALT (U/L) | 3.6 | 3.6 |
| TG (mmol/L) | 1.6 | 1.1 |
| LDL-C (mmol/L) | 4.9 | 1 |
| TC (mmol/L) | 5.4 | NA |
| BUN (mmol/L) | 1.2 | 1.1 |
| Scr (umol/L) | 1.8 | 1.8 |
| FPG (mmol/L) | 1.1 | 1.1 |

**Table S2A** The baseline characterization of males based on the inflection point of HDL-C

| HDL-C (mmol/L) | <1.540 | ≥1.540 | P-value |
| --- | --- | --- | --- |
| Participants | 8198 | 1808 |  |
| Age(years) | 50.278 ± 13.523 | 51.283 ± 13.677 | 0.004 |
| Smoking status |  |  | 0.016 |
| Current-smoker | 2843 (34.679%) | 563 (31.139%) |  |
| Ex-smoker | 522 (6.367%) | 123 (6.803%) |  |
| Never-smoker | 4833 (58.953%) | 1122 (62.058%) |  |
| Drinking status |  |  | <0.001 |
| Current-drinker | 436 (5.318%) | 146 (8.075%) |  |
| Ex-drinker | 1936 (23.616%) | 433 (23.949%) |  |
| Never- drinker | 5826 (71.066%) | 1229 (67.976%) |  |
| Family history of diabetes |  |  | 0.005 |
| No | 8018 (97.804%) | 1787 (98.838%) |  |
| Yes | 180 (2.196%) | 21 (1.162%) |  |
| SBP (mmHg) | 128.292 ± 16.730 | 129.944 ± 17.764 | <0.001 |
| DBP (mmHg) | 79.901 ± 10.917 | 80.287 ± 11.169 | 0.176 |
| BMI (kg/m^2^) | 25.535 ± 3.098 | 24.536 ± 3.239 | <0.001 |
| AST (U/L) | 27.479 ± 12.602 | 27.335 ± 12.999 | 0.661 |
| ALT (U/L) | 32.441 ± 25.629 | 30.197 ± 24.532 | <0.001 |
| TG (mmol/L) | 2.025 ± 1.497 | 1.718 ± 1.589 | <0.001 |
| LDL-C (mmol/L) | 2.863 ± 0.705 | 3.118 ± 0.686 | <0.001 |
| TC (mmol/L) | 4.921 ± 0.912 | 5.450 ± 0.879 | <0.001 |
| BUN (mmol/L) | 5.127 ± 1.220 | 5.240 ± 1.269 | <0.001 |
| Scr (umol/L) | 80.631 ± 13.043 | 79.608 ± 12.355 | 0.002 |
| FPG (mmol/L) | 5.969 ± 0.324 | 5.968 ± 0.333 | 0.843 |

Values are n (%) or mean ± SD

HDL-C: high-density lipoprotein cholesterol; SBP: systolic blood pressures; DBP: diastolic blood pressures; BMI: body mass index; AST: aspartate aminotransferase; ALT: alanine aminotransferase; LDL-C: low-density lipoprotein cholesterol; TC: total cholesterol; TG: triglycerides; Scr: serum creatinine; BUN: blood urea nitrogen; FPG: fasting plasma glucose

**Table S2B** The baseline characterization of females based on the inflection point of HDL-C

| HDL-C (mmol/L) | <1.620 | ≥1.620 | P-value |
| --- | --- | --- | --- |
| Participants | 4094 | 1320 |  |
| Age(years) | 51.231 ± 13.045 | 53.464 ± 13.435 | <0.001 |
| Smoking status |  |  | 0.016 |
| Current-smoker | 2843 (34.679%) | 563 (31.139%) |  |
| Ex-smoker | 522 (6.367%) | 123 (6.803%) |  |
| Never-smoker | 4833 (58.953%) | 1122 (62.058%) |  |
| Drinking status |  |  | <0.001 |
| Current-drinker | 436 (5.318%) | 146 (8.075%) |  |
| Ex-drinker | 1936 (23.616%) | 433 (23.949%) |  |
| Never- drinker | 5826 (71.066%) | 1229 (67.976%) |  |
| Family history of diabetes |  |  | 0.005 |
| No | 8018 (97.804%) | 1787 (98.838%) |  |
| Yes | 180 (2.196%) | 21 (1.162%) |  |
| SBP (mmHg) | 125.755 ± 18.664 | 124.647 ± 19.448 | 0.064 |
| DBP (mmHg) | 76.058 ± 11.066 | 74.964 ± 11.182 | 0.002 |
| BMI (kg/m^2^) | 24.197 ± 3.409 | 22.860 ± 3.198 | <0.001 |
| AST (U/L) | 23.862 ± 10.116 | 23.704 ± 8.606 | 0.609 |
| ALT (U/L) | 21.530 ± 16.578 | 19.294 ± 12.965 | <0.001 |
| TG (mmol/L) | 1.599 ± 1.173 | 1.211 ± 0.892 | <0.001 |
| LDL-C (mmol/L) | 2.896 ± 0.732 | 3.209 ± 0.735 | <0.001 |
| TC (mmol/L) | 4.941 ± 0.948 | 5.566 ± 0.930 | <0.001 |
| BUN (mmol/L) | 4.702 ± 1.205 | 4.865 ± 1.260 | <0.001 |
| Scr (umol/L) | 59.196 ± 11.825 | 59.269 ± 12.471 | 0.847 |
| FPG (mmol/L) | 5.930 ± 0.309 | 5.909 ± 0.296 | 0.030 |

**Table S3A** The baseline characterization based on SBP

| SBP (mmHg) | SBP<140 | SBP≥140 | P-value |
| --- | --- | --- | --- |
| Participants | 7688 | 2318 |  |
| BMI (kg/m^2^) | 25.131 ± 3.084 | 26.097 ± 3.239 | <0.001 |

**Table S3B** The baseline characterization based on smoking status

| Smoking status | Current-smoker | Ex-smoker | Never-smoker | P-value |
| --- | --- | --- | --- | --- |
| Participants | 3414 | 650 | 11356 |  |
| BMI (kg/m^2^) | 25.472 ± 3.134 | 25.506 ± 3.200 | 24.604 ± 3.347 | <0.001 |

**Table S4** Relationship between HDL-C levels and reversion to normoglycemia from Pre-DM in different models

| Exposure | Model 3 (HR,95% CI, P) | Model 4 (HR,95% CI, P) |
| --- | --- | --- |
| HDL-C | 1.904 (1.765, 2.055) <0.001 | 1.897 (1.757, 2.047) <0.00001 |
| HDL-C (quartiles) |  |  |
| Q1 | ref | ref |
| Q2 | 1.201 (1.117, 1.290) <0.001 | 1.198 (1.115, 1.287) <0.001 |
| Q3 | 1.475 (1.373, 1.586) <0.001 | 1.469 (1.367, 1.579) <0.001 |
| Q4 | 1.739 (1.615, 1.873) <0.001 | 1.732 (1.608, 1.865) <0.001 |
| P for trend | <0.001 | <0.001 |

Model 3: we adjusted for DBP, SBP, BMI, gender, age, drinking status, family history of diabetes, smoking status, FPG, AST, BUN, LDL-C, Scr, and TG, and did not adjust for ALT.

Model 4: we adjusted for DBP, SBP, BMI, gender, age, drinking status, family history of diabetes, smoking status, FPG, BUN, ALT, LDL-C, Scr, and TG , and did not adjust for AST.

HR: hazard ratios; CI: confidence interval; Ref: reference; GAM: generalized additive mode; HDL-C: high-density lipoprotein cholesterol
